# Determination of the Enantiomerization Barrier of Midazolam in Aqueous Conditions by Electronic Circular Dichroism and Dynamic Enantioselective HPLC/UHPLC

**DOI:** 10.3390/molecules30051108

**Published:** 2025-02-28

**Authors:** Francesca Romana Mammone, Daniele Sadutto, Eleonora Antoniella, Marco Pierini, Roberto Cirilli

**Affiliations:** 1 Centre for the Control and Evaluation of Medicines, Chemical Medicines Unit, Istituto Superiore di Sanità, Viale Regina Elena 299, 00161 Rome, Italy; francescaromana.mammone@uniroma1.it (F.R.M.); daniele.sadutto@iss.it (D.S.); eleonora.antoniella@iss.it (E.A.); 2 Department of Drug Chemistry and Technology, “Sapienza” University of Rome, 00185 Rome, Italy

**Keywords:** Chiralpak IG-3, midazolam, enantioselective dynamic HPLC, electronic circular dichroism, enantiomerization barrier

## Abstract

Midazolam is a benzodiazepine that is utilized for the induction of anesthesia and the facilitation of procedural sedation. Despite the absence of stereogenic centers, the non-planar seven-membered ring devoid of reflection symmetry elements confers planar stereogenicity to the molecule. Due to the rapid conformational inversion of the *Rp* and *Sp* enantiomers, which occurs via a simple ring flip, high-performance liquid chromatography (HPLC) enantiomeric separation is restricted to sub-room temperature conditions. In this study, the energy barriers for the racemization of midazolam at five distinct temperatures and in acetonitrile/water mixtures were determined by monitoring the decay of the circular dichroism signal at a specific wavelength over time. The kinetic and thermodynamic data obtained were compared with those determined by dynamic enantioselective high-performance liquid chromatography using the Chiralpak IG-3 chiral stationary phase, which contains the amylose tris(3-chloro-5-methylphenylcarbamate) as the selector. The temperature-dependent dynamic HPLC of midazolam was carried out at the same temperatures and with the same aqueous mixtures used in parallel kinetic off-column experiments. To simulate dynamic chromatographic profiles, a lab-made computer program based on a stochastic model was utilized. The results indicated that the moderate influence of the stationary phase resulted in a slight increase in the activation barriers, which was more pronounced as the time spent in the column increased. This phenomenon was found to be mitigated when switching from a 250 mm × 4.6 mm, 3 µm, Chiralpak IG-3 column to a 50 mm × 4.6 mm, 1.6 µm, Chiralpak IG-U UHPLC column. The outcomes obtained under UHPLC conditions were found to be more closely aligned with those obtained through the ECD technique, with a discrepancy of only 0.1 kcal/mol or less, indicating a high degree of concordance between the two methods.

## 1. Introduction

The concepts of molecular chirality and stereochemistry are fundamental to numerous disciplines, including pharmaceutical chemistry. As time progresses, the importance of chirality in a plethora of applications involving chiral drugs has been increasingly recognized, particularly in the context of interactions between biological systems and enantiomers of pharmaceutical agents [1]. The biological properties of the two enantiomers of a compound often exhibit marked differences in terms of pharmacodynamics and pharmacokinetic properties, rendering the susceptibility of these compounds to racemization in aqueous solutions a crucial piece of information. The process of racemization can have detrimental effects on the efficacy and safety of pharmaceuticals. It can reduce the dose of the active compound, leading to suboptimal therapeutic outcomes. Additionally, it can result in exposure to potentially harmful compounds, even when only the safer enantiomer has been taken [2]. Racemization is also a crucial aspect of drug discovery, as it can determine the economic viability of chiral switching. Chiral switching refers to the replacement of a racemate, which is no longer protected by a patent, with an improved single enantiomer that still has patent protection.

In consideration of these factors, concerns have been raised regarding the racemization process, particularly under conditions that bear resemblance to physiological conditions.

The Food and Drug Administration (FDA) and other regulatory bodies have established requirements for the submission of definitive data concerning the configurational stability of chiral drugs [3].

In recent years, it has become evident that significant advancements have been made in the comprehension of racemization across various disciplines. These strategies have been employed to either promote (e.g., for the study of deracemization or dynamic kinetic resolution processes) or avoid racemization induced by chemists and nature [4,5,6]. Nevertheless, these studies require improvement and updating, particularly in cases where kinetic data are either absent or limited in availability. In this context, it is beneficial to consider the relationship between microscopic (molecular) processes and macroscopic racemization. Racemization can be defined as a statistical, macroscopic, and irreversible process. Essentially, this process involves converting half of an initial enantiopure quantity of a given compound into its enantiomer. The process is deemed complete once the enantiomeric excess (e.e.) of the analyzed sample is diminished to 0%.

The process of racemization is a macroscopic phenomenon, as the racemization of a single molecule is not a feasible occurrence. Enantiomerization is associated with racemization being a microscopic process that involves the reversible conversion of a single molecule of an enantiomer into its optical antipode. The repeated occurrence of enantiomerization ultimately culminates in racemization. In a system undergoing enantiomerization, the observed rate constant, (*krac*), for achieving dynamic equilibrium is the sum of the two enantiomerization rate constants, forward and upward, (*ke*), which are equal and define the corresponding rate constant for racemization (*krac* = 2*ke*).

In recent decades, computational studies have played a pivotal role in elucidating the racemization mechanism of stereolable compounds and in predicting their rate and barriers of racemization [7,8]. Typically, mechanistic studies are supported by experimental kinetic data obtained through effective techniques such as dynamic nuclear magnetic resonance (NMR) [9,10], electronic circular dichroism (ECD) [11,12], dynamic enantioselective gas chromatography (GC) [13,14,15,16], dynamic high-performance liquid chromatography (HPLC) [17,18,19,20,21,22,23,24], and dynamic capillary electrophoresis (CE) [25,26].

**Figure 1:**
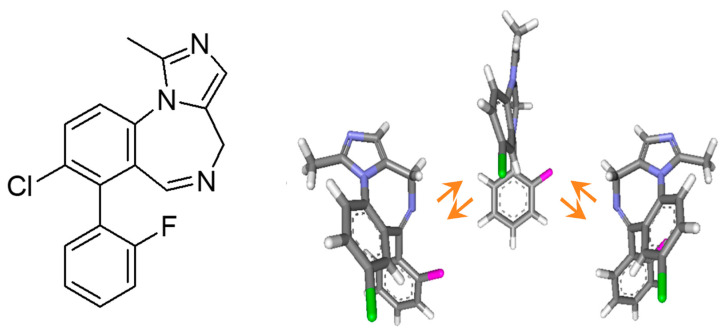
Structure of midazolam (**left**) and polytube model of the interconversion process of its conformational enantiomers (**right**).

In this context, midazolam, a benzodiazepine medication employed in anesthesia and procedural sedation, is an appropriate candidate for racemization studies. Similar to other benzodiazepines, midazolam has a non-planar structure, which resembles the cycloheptatriene boat conformation (Figure 1). The existence of a non-planar seven-membered ring devoid of reflection symmetry elements gives rise to planar stereogenicity. The conformational inversion of the *Rp* and *Sp* enantiomers, which occurs via a simple ring flip, is a rapid process at room temperature (Figure 1) as demonstrated in a previous work by dynamic HPLC followed by computer simulation of experimental HPLC traces [27].

Midazolam exemplifies a noteworthy case of stereochemical unstable drugs and drug-like molecules, exhibiting energy barriers of approximately 20 kcal/mol and half-life times that range from minutes to fractions of seconds. Although this rapid process hinders the testing of enantiomers at physiological temperatures, the dynamic stereochemical behavior exhibited by these types of molecules remains a subject of interest, as they demonstrate conformational enrichment at binding sites, such as serum proteins and protein receptors [28,29].

The dynamic HPLC approach used in midazolam enantiomerization barrier determination entails the selection of an experimental dynamic chromatogram exhibiting a plateau between the two enantiomeric resolved peaks for simulation purposes [27]. A lab-made program based on a stochastic model is used to simulate the dynamic HPLC profiles of midazolam. The program employs separation and retention factors, along with efficiency data derived directly from the experimental chromatogram, as input parameters. Once the discrepancy between the experimental and computed HPLC plots has reached a minimum value, the computational procedure returns the apparent rate constants (*kapp*) for the interconversion of the two enantiomers, designated as *kapp_1→2_* and *kapp_2→1_* (where the subscript numbers correspond to the forward process, which converts the initially eluted enantiomer into the second, designated as *kapp_1→2_*, and the backward process, designated as *kapp_2→1_*). The apparent rate constants represent a weighted average value for the interconversion occurring in both the mobile (*km*) and stationary phases (*ks*) [17,18,19,20,21,22,23,24,30]. The two rate constants are, in principle, distinct due to the potential for the stationary phase to exert a perturbing effect on the reversible process, which differs for two eluting enantiomers that spend different times in the adsorbed state. This effect may be either retarding or activating [31]. The apparent enantiomerization barriers ∆G^#^*app* for the forward and upward on-column interconversion of enantiomers of midazolam obtained by dynamic HPLC (DHPLC) on the Chiralpak IA chiral stationary phase (CSP) and computer simulation are 20.17 kcal/mol and 20.83 kcal/mol in normal-phase eluent condition and 20.26 kcal/mol and 20.49 kcal/mol in reversed-phase (RP) mode [27].

In the absence of data on the influence of chiral stationary phase disturbance on the enantiomerization barrier value, this study aims to subject midazolam to a comprehensive kinetic analysis in aqueous conditions using independent dynamic HPLC/UHPLC (i.e., DHPLC/DUHPLC) and off-column ECD measurements. In particular, we explore the potential advantages of utilizing a commercially available sub–2 μm immobilized polysaccharide (Chiralpak IG-U) column to ensure the accuracy and reliability of the theoretical calculations of the enantiomerization barrier through enantioselective dynamic chromatography.

## 2. Results and Discussion

### 2.1. Enantioseparations with Acetonitrile/Water Mixtures

**Figure 2:**
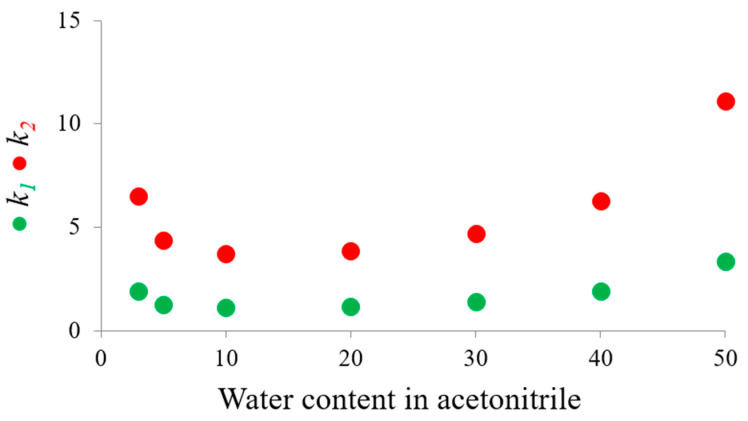
Plots of the retention factors (*k*_1_ and *k*_2_) of the enantiomers of midazolam as a function of the water content in the acetonitrile–aqueous mode. Chromatographic conditions: column, Chiralpak IG-3 (250 mm × 4.6 mm, 3 μm); temperature, 5 °C; flow rate, 1.0 mL/min (from 5 to 30% of water in acetonitrile) and 0.7 mL/min (40% and of 50% water in acetonitrile); detection, UV at 254 nm.

In this study, the chromatographic behavior of midazolam on the 250 mm × 4.6 mm Chiralpak IG-3 column, containing 3 μm silica particles as packing material on which amylose tris(3-chloro-5-methylphanylcarbamate) has been immobilized, was evaluated, with mobile phases consisting of a mixture of acetonitrile and increasing percentages of water (i.e., 3, 5, 10, 20, 30, 40, 50%). The column temperature was fixed at 5 °C to reduce the competitive effect of on-column enantiomerization on the enantioseparation process. Figure 2 shows the graphs obtained plotting the retention factors of the midazolam enantiomers against increasing water content in the binary mobile phases.

The shape of the retention maps allows the definition of two distinct regions, which are characteristic of two different retention mechanisms. These regions are separated by a crossover water level, which occurs at approximately 15%. In the region on the left, from 3% to 15% water, the retention of the enantiomers demonstrates a decrease with an increase in water, whereas in the range on the right, from 15% to 50%, it exhibits a progressive increase. The U-shaped nature of these plots confirms the presence of polar active sites within the chlorinated polysaccharide-based CSPs, which are responsible for the HILIC retention mechanism in the presence of low water contents mixed with acetonitrile [32,33,34]. The RP retention mechanism overcomes the HILIC retention behavior when the water content exceeds the balancing mobile phase composition. In the range of mobile phase compositions investigated, the enantioseparation factor remains practically constant at a high value of about 3.3.

The dual and competitive HILIC-RP retention mechanism and the resulting curvature of the retention maps are particularly suitable for the analysis of stereolable pharmaceutical compounds.

The advantages are twofold. First, it is possible to study the effect of water on the enantiomerization barrier (see next sections for details) and to evaluate aqueous environments that closely mimic the in vivo situation. Second, it is possible to limit the retention times of the enantiomers in the column, thereby reducing the impact of the secondary enantiomerization process that competes with the primary adsorption/desorption process.

### 2.2. Determination of Enantiomerization Barriers by Electronic Circular Dichroism

In consideration of the proportionality between ellipticity and enantiomeric excess (e.e.) and the first-order kinetic of the conformational flip of midazolam, it can be inferred that the concentration has no impact on the decay in ellipticity over time [11]. Consequently, the decay of the e.e. adheres to the same fundamental principles as those governing the ECD signal. A linear regression analysis applied to the plot of the natural logarithm of the ellipticity versus time (ln(e.e.) vs. time) yields the rate constant of racemization at a given temperature.

**Figure 3:**
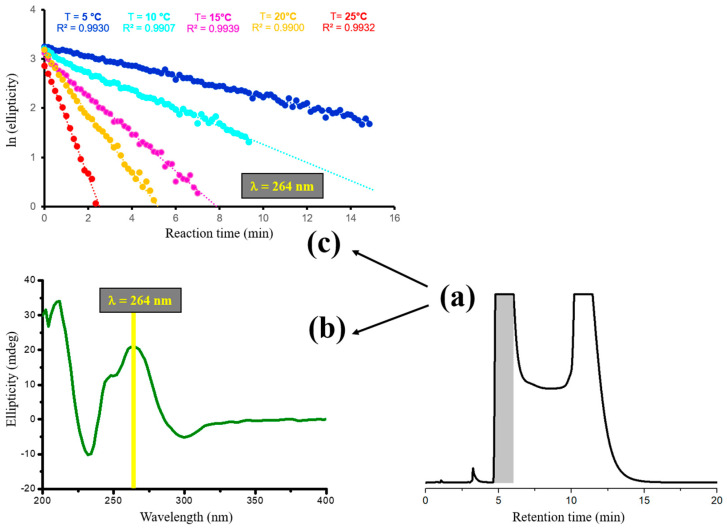
Off-column racemization of midazolam monitored by enantioselective ECD methods. (**a**) HPLC enantioseparation of 0.33 mg of midazolam. Chromatographic conditions: column, Chiralpak IG-3 (250 mm × 4.6 mm, 3 μm); temperature, 5 °C; mobile phase, acetonitrile/water 80:20 (*v*/*v*); flow rate, 1.3 mL/min; detection, UV at 280 nm. (**b**) ECD spectrum of the first eluted enantiomer collected under the same conditions as in (**a**) and recorded at 5 °C; (**c**) ECD signal decay at 264 nm of the first eluted enantiomer monitored at 5–25 °C.

In contrast with the kinetic determination obtained by dynamic enantioselective HPLC, the off-column ECD approach requires the initial isolation of the enriched forms from enantioselective HPLC separation runs performed under conditions that minimize competitive on-column enantiomerization. This is followed by the monitoring of the racemization process within the cuvette of the ECD instrument, which is thermostated at a specified temperature. Figure 3 depicts a representative configuration of experiments employed in kinetic determination pertinent to the interconversion of the conformational enantiomers of midazolam.

The enantioseparation of midazolam was accomplished using the Chiralpak IG-3 (250 mm × 4.6 mm, 3 μm) column. The acetonitrile/water mixtures, with a ratio of 90:10, 80:20, 70:30, 60:40, and 50:50 (*v*/*v*), were utilized as mobile phases at a temperature of 5 °C. The flow rate was set at 1.0 mL/min for the initial three elution conditions and 0.7 mL/min for the remaining ones. For all elution conditions, two well-resolved peaks were observed, corresponding to the conformational enantiomers of midazolam. Additionally, a plateau was noted between the enantiomeric peaks. The characteristic deviation from the usual chromatographic profile was indicative of the stereolability of midazolam and the simultaneous occurrence of on-column interconversion of two enantiomers during the same time frame as the separation process [17,18,19,20,21,22,23,24,30].

However, the low temperature and flow rate used for the resolution of midazolam were sufficient to minimize competitive and perturbative on-column enantiomerization and allow for the isolation of an enriched form of the first eluting enantiomer, which was then rapidly submitted to ECD measurements. In order to obtain an ECD signal of sufficient intensity for the purpose of studying the ECD decay over time, the wavelength of 264 nm, which corresponds to the maximum of the previously recorded ECD spectrum, was employed.

Table 1 presents the calculated energy barrier values obtained from off-column measurements carried out at temperatures of 5 °C, 10 °C, 15 °C, 20 °C, and 25 °C. The presence of acetonitrile/water mixtures with varying compositions was investigated, specifically 90:10, 80:20, 70:30, 60:40, and 50:50 (*v*/*v*).

The data analysis yielded the following conclusions:

i. A change in water content from 10 to 50% in acetonitrile, at equal temperatures, resulted in modest changes in the ΔG^#^ barriers (the ΔΔG^#^ values ranged from 0.13 to 0.29 kcal/mol).
ii. For each reaction mixture, at different conditions of temperature, the enantiomerization energy barriers were found to be highly similar, with a maximum absolute discrepancy of 0.20 kcal/mol. This provided compelling evidence that the first-order enantiomerization process was scarcely influenced by the entropy term, which was found to be for all conditions investigated lower than 8.0 e.u.
iii. The observed stereolability of midazolam was consistent with the results reported elsewhere [27], which were obtained by DHPLC on the amylose-based Chiralpak IA (250 mm × 4.6 mm, 5 µm) column. The use of acetonitrile/water 70:30 (*v*/*v*) as the mobile phase at 15 °C resulted in a discrepancy of only 0.25 kcal/mol (i.e., 20.13 kcal/mol vs. 20.38 kcal/mol), which may be attributed to a slight perturbation effect exerted by CSP on on-column interconversion.
iv. At 25 °C, the half-life of the racemization process was found to be less than one minute in all conditions investigated.

### 2.3. Determination of Enantiomerization Barriers by Dynamic Enantioselective Chromatography

An independent dynamic enantioselective HPLC analysis of midazolam was performed on the Chiralpak IG-3 (250 mm × 4.6 mm, 3 µm) column, employing the identical acetonitrile/water mixtures utilized in ECD experiments (90:10, 80:20, 70:30, 60:40, and 50:50, *v*/*v*) and the same temperatures (5 °C, 10 °C, 15 °C, 20 °C, and 25 °C).

**Figure 4:**
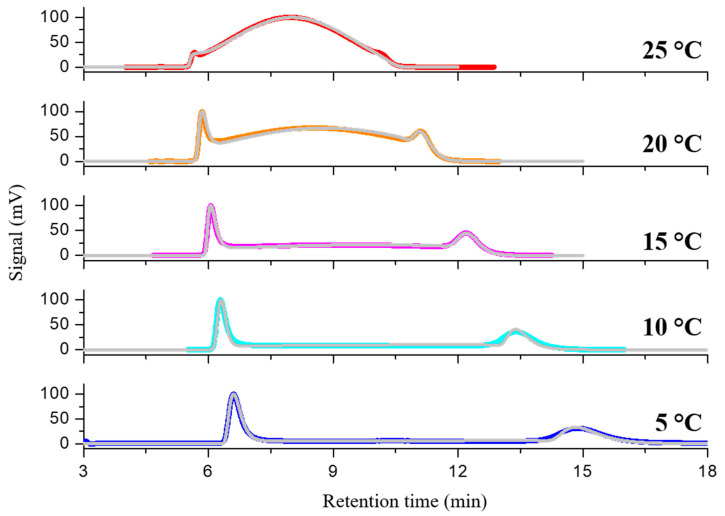
An illustrative example of dynamic enantioselective HPLC determinations pertinent to the enantiomerization process of midazolam from 5 to 25 °C. The simulated dynamic chromatograms (gray traces) are superimposed on the corresponding experimental ones (colored traces). Chromatographic conditions: column, Chiralpak IG-3 (250 mm × 4.6 mm, 3 μm); mobile phase, acetonitrile/water 80:20 (*v*/*v*); flow rate, 1 mL/min; detection, UV at 254 nm.

Figure 4 illustrates the dynamic chromatograms obtained with the acetonitrile/water mixture 90:10 (*v*/*v*) across a temperature range of 5–25 °C. As can be observed, the height of the plateau exhibited a gradual increase with rising temperature, resulting from a faster on-column interconversion rate. At 25 °C, the enantiomeric peaks exhibited near-complete coalescence. It is noteworthy that, with the mobile phases acetonitrile/water 60:40 (*v*/*v*) and acetonitrile/water 50:50 (*v*/*v*), the flow rate was adjusted from 1 to 0.7 mL/min due to the higher viscosity, which consequently resulted in increased backpressure on the column. The process of enantiomerization was observed to become more competitive in terms of enantiomer separation as the time spent in the column was increased. This resulted in a reduction of the coalescence temperature to approximately 20 °C.

The dynamic HPLC traces generated by varying the column temperature and mobile phase composition were simulated using the lab-made Auto DHPLC y2k computer program, which employs a stochastic model. Figure 4 illustrates a selected set of simulated dynamic chromatograms (grey traces) superimposed on the corresponding experimental HPLC traces (colored traces).

The apparent rate constants for the forward and backward interconversion processes, *kapp_1→2_* and *kapp_2→1_*, respectively, and the corresponding energy barriers, calculated by the Eyring equation assuming a unitary transmission coefficient, are provided in Table 2.

**Figure 5:**
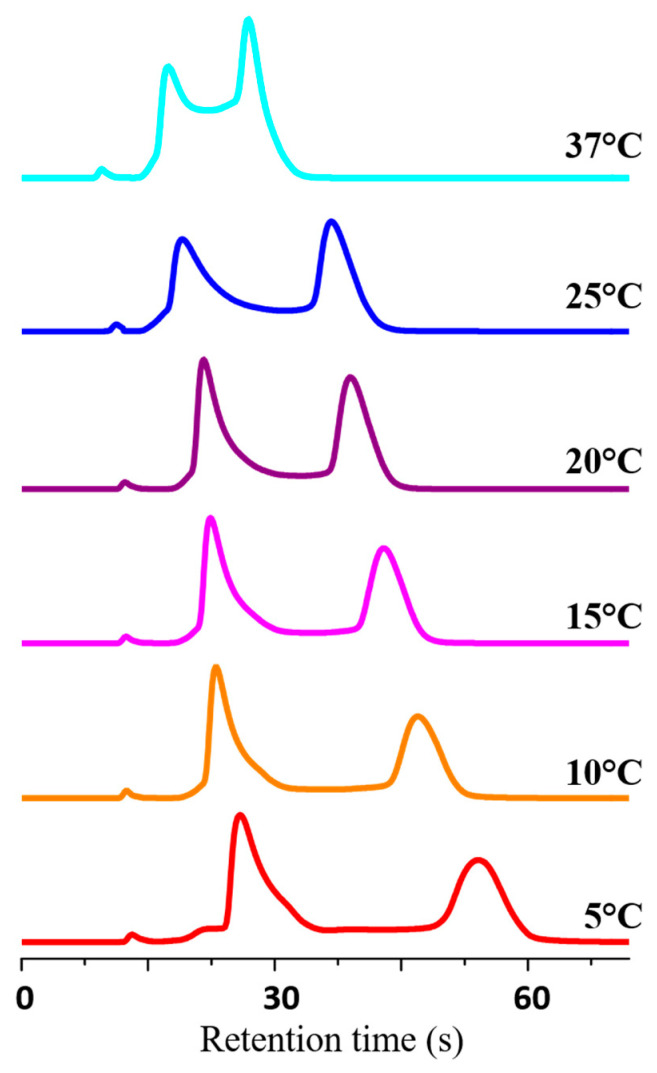
UHPLC traces for the enantioseparation of midazolam from 5 to 37 °C. Chromatographic conditions: column, Chiralpak IG-U (50 mm × 3.0 mm, 1.6 μm); mobile phase, acetonitrile/water 90:10 (*v*/*v*); flow rate, 1.5 mL/min; detection, UV at 254 nm.

A comparison of the kinetic data obtained by DHPLC indicated that, in all investigated conditions, there was a minimal discrepancy between *kapp_1→2_* and *kapp_2→1_*, with *kapp_1→2_* consistently exhibiting a higher value than *kapp_2→1_*. The discrepancies in the corresponding ΔG^#^ values increased with a reduction in temperature within a given mobile phase composition and an increase in water content. The maximum divergence in the ΔG^#^ amounts (∆G^#^*_app1→2_* and ∆G^#^*_app2→1_*) was 0.56 kcal/mol, which was observed at 5 °C with the mobile phase acetonitrile/water 50:50 (*v*/*v*). This suggests that the interconversion from the second eluted enantiomer, which formed a more structured complex with the CSP, to the first eluted enantiomer occurred at a slower rate than the reverse process. In other words, the interconversion occurring in the adsorbed state was more impeded than in the liquid phase, which resulted in the CSP exhibiting a slight inhibitory effect on the ring flip process. The analysis of the kinetic constants indicated that, as the degree of retention of the enantiomer increased (as illustrated in Figure 2, which depicts the trend of the retention factor values as the water concentration in the mobile phase changed), so did the interaction with the stationary phase. Consequently, the retarding effect of CSP on the conformational interconversion process became more pronounced. It would therefore be advantageous to limit the residence time of the enantiomers within the column. An effective approach would be to use a short UHPLC column packed with 1.6 μm silica particles containing amylose tris(3-chloro-5-methylphanylcarbamate) as a selector (the commercial name is Chiralpak IG-U), namely the same as the Chiralpak IG-3 CSP. In this study, the 50 mm × 3.0 mm Chiralpak IG-U column was used with acetonitrile/water 90:10 (*v*/*v*) mobile phase at temperatures of 5 °C, 10 °C, 15 °C, 20 °C, 25 °C, and 37 °C. The flow rate was set at 1.5 mL/min. As shown in Figure 5, the use of the 50 mm × 3.0 mm Chiralpak IG-U column packed with 1.6 μm diameter particles allowed the rapid elution of two resolved enantiomers without reaching peak coalescence due to the enantiomerization process. At the physiological temperature of 37 °C, despite the racemization process having a half-life of only 24 s, the dynamic cluster was eluted with sufficient rapidity (i.e., within 30 s) that the two enantiomeric peaks, connected by a plateau at approximately half height, were still clearly distinguishable.

Table 3 shows a comparative analysis of the supplementary kinetic data pertaining to the on-column interconversion of midazolam, as obtained through dynamic UHPLC and HPLC techniques. As can be seen, the disparity in the corresponding ΔG^#^ amounts was found to be in the order of 0.1 kcal/mol or less, indicating a relatively minor impact on the overall interconversion process. Specifically, while all the ∆∆G^#^ values obtained by considering barriers estimated through the dynamic HPLC approach resulted always slightly negative (that is, with respect to the ΔG^#^_ECD_ data, at any temperature the ΔG^#^_app_ values were slightly overestimated, with an averaged ∆∆G^#^ difference amounting to −0.138 kcal/mol and with a medium percentage effect on the half-life times (expressed in minutes) of +27.4%), the equivalent ∆∆G^#^ data obtained making reference to the barriers assessed by simulation of dynamic UPLC traces (DHUPLC) resulted much closer to the ΔG^#^_ECD_ data (the averaged ∆∆G^#^ difference, in this case, was lightly positive and amounted to only 0.058 kcal/mol, with a medium percentage effect on the half-life times (expressed in minutes) assessed at −9.4%).

## 3. Materials and Methods

### 3.1. Materials

Midazolam was purchased from Sigma-Aldrich (Milan, Italy).

Chromatographic enantioseparations were performed by using the commercially available stainless steel Chiralpak^®^ IG-3 (250 mm × 4.6 mm, 3 μm) and Chiralpak^®^ IG-U (50 mm × 4.6 mm, 1.6 μm) columns (Chiral Technologies Europe, Illkirch, France).

### 3.2. Instruments and Chromatographic Conditions

In studies of racemization conducted in off-column mode, solutions containing 1.0 mg of midazolam in 0.3 mL of acetonitrile/tetrahydrofuran mixture 1:3 (*v*/*v*) were prepared. Ten microliters of the sample solutions were injected onto the 250 mm × 4.6 mm Chiralpak IG-3 column. The analytical HPLC apparatus comprised a Perkin-Elmer pump (LC 2000 series) (Norwalk, CT, USA), a Rheodyne injector (Cotati, CA, USA), a 50 μL sample loop, a PerkinElmer LC 101 oven, and a Waters 484 UV detector (Waters Corporation, Milford, MA, USA). The signal was acquired and processed using Clarity TM 7.1.0.151 software from DataApex (Prague, Czech Republic). The fraction containing the first eluting enantiomer was rapidly submitted to ECD analysis to monitor the e.e. decay over time.

Dynamic UHPLC analyses were performed on the Waters ACQUITY ARC System (Milford, MA, USA). This instrument included a quaternary pumping system with a maximum flow rate of 2 mL/min, an autosampler with an injection loop volume of 5 μL (used in partial loop mode), a 2998 photodiode array detector, and a column oven. Data acquisition was performed using Empower software (version 3.0).

For dynamic chromatographic analysis, freshly prepared standards for chromatographic analysis were obtained by dissolving midazolam in acetonitrile. Injection volumes were 10–30 μL for dynamic HPLC analysis and 2.0 μL for dynamic UHPLC analysis.

The ECD spectrum of the first eluted enantiomer of midazolam was measured in a 0.1 cm path length quartz cell at 25 °C using a Jasco model J-700 spectropolarimeter (Jasco, Tokyo, Japan). The intensities were expressed as ellipticity values (mdeg).

### 3.3. Simulation of Dynamic Chromatograms

Auto-DHPLC-y2k lab-made software was used to simulate experimental dynamic chromatograms. The program implements both stochastic and theoretical plate models, as outlined in reference [24], and is capable of accounting for all types of first-order interconversion, as along with tailing effects.

## 4. Conclusions

The energy barriers for enantiomerization of midazolam were meticulously assessed through the implementation of off-column racemization kinetics and dynamic HPLC/UHPLC techniques, both of which were founded upon the enantiomer separation capability of chiral stationary phases based on amylose tris(3-chloro-5-methylphanylcarbamate). This CSP demonstrated remarkable versatility and efficiency, yielding highly satisfactory enantioseparation results across a broad range of aqueous elution modes, even at relatively low temperatures necessary for the isolation of the conformationally labile antipodes of midazolam. It was demonstrated that the moderate influence of the stationary phase resulted in a slight increase in the activation barriers, which was more pronounced as the time spent in the column increased (determinations performed by DHPLC). The phenomenon was suppressed when a switch was made from a 250 mm × 4.6 mm, 3 µm, Chiralpak IG-3 column to a 50 mm × 4.6 mm, 1.6 µm, Chiralpak IG-U UHPLC column. These findings provide empirical evidence that the employment of highly efficient sub-2-μm chiral packing for ultrafast chiral separations better supports the simulated data, thereby enhancing the accuracy and reliability of the dynamic chromatographic technique, thus suggesting that, in some cases, for kinetic studies requiring particularly high precision, a transition from the DHPLC to the DHUPLC technique may be advantageous.

## Figures and Tables

**Table 1:** Kinetic constants and activation parameters for the enantiomerization of midazolam by ECD determinations under different aqueous and temperature conditions. ^a^: t_0.5_ is the half-life of the racemization process; ^b^: ΔG^#^ data were obtained by the relevant rate constant values through the Eyring equation (transmission factor was set to 1); ^c^: errors associated with energy barrier values were lower or equal to 0.5%.

Mobile Phase	T (°C)	k (s^−1^)	t_0.5_^a^ (min)	∆G^# b,c^ (kcal/mol)	∆H^#^ (kcal/mol)	∆S^#^ (e.u.)
Acetonitrile/water 90:10	25	0.0173	0.67	20.27 ± 0.10	17.85 ± 0.09	−7.92 ± 0.40
	20	0.0122	0.95	20.12 ± 0.10		
	15	0.0062	1.86	20.15 ± 0.10		
	10	0.0038	3.04	20.07 ± 0.10		
	5	0.0019	6.08	20.08 ± 0.10		
Acetonitrile/water 80:20	25	0.0192	0.60	20.20 ± 0.10	19.23 ± 0.08	−3.28 ± 0.17
	20	0.0099	1.17	20.24 ± 0.10		
	15	0.0064	1.81	20.14 ± 0.10		
	10	0.0031	3.73	20.18 ± 0.10		
	5	0.0017	6.80	20.15 ± 0.10		
Acetonitrile/water 70:30	25	0.0158	0.73	20.32 ± 0.10	19.86 ± 0.09	−1.24 ± 0.08
	20	0.0107	1.08	20.20 ± 0.10		
	15	0.0065	1.78	20.13 ± 0.10		
	10	0.0028	4.13	20.24 ± 0.10		
	5	0.0014	8.25	20.26 ± 0.10		
Acetonitrile/water 60:40	25	0.0135	0.86	20.41 ± 0.10	18.90 ± 0.08	−4.82 ± 0.20
	20	0.0091	1.27	20.29 ± 0.10		
	15	0.0056	2.06	20.21 ± 0.10		
	10	0.0027	4.28	20.26 ± 0.10		
	5	0.0013	8.89	20.30 ± 0.10		
Acetonitrile/water 50:50	25	0.0147	0.79	20.36 ± 0.10	18.89 ± 0.07	−4.93 ± 0.21
	20	0.0080	1.44	20.36 ± 0.10		
	15	0.0050	2.31	20.28 ± 0.10		
	10	0.0024	4.81	20.33 ± 0.10		
	5	0.0014	8.25	20.26 ± 0.10		

**Table 2:** Kinetic constants and activation parameters for the enantiomerization of midazolam by dynamic HPLC determinations under different aqueous and temperature conditions. ^a^: ∆G^#^*app* is the average value between ∆G^#^*app_1→2_* and ∆G^#^*app_2→1_*.

Mobile Phase	T (°C)	*kapp_1→2_* (s^−1^)	*kapp_2→1_* (s^−1^)	∆G^#^*app_1→2_* (kcal/mol))	∆G^#^*app_2→1_* (kcal/mol)	∆G^#^*app* ^a^ (kcal/mol)
Acetonitrile/water 90:10	25	0.0083	0.0049	20.29 ± 0.10	20.61 ± 0.10	20.45 ± 0.10
	20	0.0059	0.0032	20.13 ± 0.10	20.48 ± 0.10	20.31 ± 0.10
	15	0.0036	0.0019	20.06 ± 0.10	20.44 ± 0.10	20.25 ± 0.10
	10	0.0021	0.001	20.02 ± 0.10	20.42 ± 0.10	20.21 ± 0.10
	5	0.0012	0.0006	19.95 ± 0.10	20.38 ± 0.10	20.16 ± 0.10
Acetonitrile/water 80:20	25	0.0105	0.0057	20.15 ± 0.10	20.51 ± 0.10	20.33 ± 0.10
	20	0.0059	0.0031	20.13 ± 0.10	20.51 ± 0.10	20.32 ± 0.10
	15	0.0035	0.0017	20.08 ± 0.10	20.48 ± 0.10	20.28 ± 0.10
	10	0.0023	0.0011	19.97 ± 0.10	20.39 ± 0.10	20.17 ± 0.10
	5	0.0014	0.0006	19.88 ± 0.10	20.33 ± 0.10	20.10 ± 0.10
Acetonitrile/water 70:30	25	0.0095	0.0050	20.21 ± 0.10	20.59 ± 0.10	20.40 ± 0.10
	20	0.0057	0.0029	20.16 ± 0.10	20.56 ± 0.10	20.36 ± 0.10
	15	0.0036	0.0017	20.06 ± 0.10	20.49 ± 0.10	20.27 ± 0.10
	10	0.0021	0.00096	20.00 ± 0.10	20.46 ± 0.10	20.23 ± 0.10
	5	0.0015	0.00062	19.85 ± 0.10	20.33 ± 0.10	20.09 ± 0.10
Acetonitrile/water 60:40	25	0.0081	0.0041	20.30 ± 0.10	20.70 ± 0.10	20.50 ± 0.10
	20	0.0054	0.0025	20.19 ± 0.10	20.63 ± 0.10	20.41 ± 0.10
	15	0.0031	0.0014	20.16 ± 0.10	20.62 ± 0.10	20.39 ± 0.10
	10	0.0018	0.00075	20.11 ± 0.10	20.59 ± 0.10	20.35 ± 0.10
	5	0.0010	0.00041	20.05 ± 0.10	20.55 ± 0.10	20.29 ± 0.10
Acetonitrile/water 50:50	25	-	-	-	-	-
	20	0.0044	0.0019	20.31 ± 0.10	20.79 ± 0.10	20.55 ± 0.10
	15	0.0023	0.0009	20.33 ± 0.10	20.83 ± 0.10	20.58 ± 0.10
	10	0.0015	0.0006	20.19 ± 0.10	20.73 ± 0.10	20.46 ± 0.10
	5	0.0010	0.0004	20.03 ± 0.10	20.59 ± 0.10	20.31 ± 0.10

**Table 3:** Comparison of apparent kinetic constants (*kapp_1→2_*, *kapp_2→1_*) and apparent energy barriers (ΔG^#^app) of midazolam obtained by simulating dynamic HPLC and HUPLC traces by a stochastic model. ^a^ ΔΔG^#^: ΔG^#^_ECD_—ΔG^#^*_app_* (ΔG^#^_ECD_: energy barrier obtained from ECD experiments). ^b^ Chromatographic conditions: column, Chiralpak IG-3 (250 mm × 4.6 mm; 3 μm); mobile phase, acetonitrile/water 90:10 (*v*/*v*); flow rate, 1.0 mL/min; detector UV/CD at 254 nm. ^c^ Chromatographic conditions: column, Chiralpak IG-U (50 mm × 3.0 mm; 1.6 μm); mobile phase, acetonitrile/water 90:10 (*v*/*v*); flow rate, 1.5 mL/min; detector UV/CD at 254 nm.

Column	T (°C)	*kapp_1→2_* (min^−1^)	*kapp_2→1_* (min^−1^)	∆G^#^*app* (kcal/mol)	∆∆G^# a^ (kcal/mol)
Chiralpak IG-3 ^b^	25	0.500	0.291	20.45	−0.18
Chiralpak IG-U ^c^		0.820	0.420	20.19	0.08
Chiralpak IG-3	20	0.358	0.198	20.31	−0.19
Chiralpak IG-U		0.548	0.304	20.06	0.06
Chiralpak IG-3	15	0.218	0.113	20.25	−0.10
Chiralpak IG-U		0.260	0.135	20.14	0.01
Chiralpak IG-3	10	0.126	0.061	20.21	−0.14
Chiralpak IG-U		0.201	0.070	19.96	0.11
Chiralpak IG-3	5	0.074	0.034	20.16	−0.08
Chiralpak IG-U		0.093	0.040	20.05	0.03

## Data Availability

Data are contained within the article.
